# Expert opinion on the diagnostic odyssey and management of late-onset Pompe disease: a neurologist's perspective

**DOI:** 10.3389/fneur.2023.1095134

**Published:** 2023-05-17

**Authors:** Sevim Erdem Ozdamar, Ayse Filiz Koc, Hacer Durmus Tekce, Dilcan Kotan, Ahmet Hakan Ekmekci, Ihsan Sukru Sengun, Ayse Nur Yuceyar, Kayihan Uluc

**Affiliations:** ^1^Department of Neurology, Hacettepe University Faculty of Medicine, Ankara, Türkiye; ^2^Department of Neurology, Cukurova University Faculty of Medicine, Adana, Türkiye; ^3^Department of Neurology, Istanbul Faculty of Medicine, Istanbul University, Istanbul, Türkiye; ^4^Department of Neurology, Sakarya University Faculty of Medicine, Sakarya, Türkiye; ^5^Department of Neurology, Selcuk University Faculty of Medicine, Konya, Türkiye; ^6^Department of Neurology, Dokuz Eylul University Faculty of Medicine, Izmir, Türkiye; ^7^Department of Neurology, Ege University Faculty of Medicine, Izmir, Türkiye; ^8^Department of Neurology, Marmara University School of Medicine, Istanbul, Türkiye

**Keywords:** late-onset Pompe disease, diagnostic delay, diagnostic algorithm, index of suspicion, treatment, outcome

## Abstract

This consensus statement by a panel of neurology experts aimed to provide a practical and implementable guidance document to assist clinicians with the best clinical practice in terms of diagnosis, treatment, and monitoring of late-onset Pompe disease (LOPD). The participating experts consider the clinical suspicion of LOPD by the physician to be of utmost importance in the prevention of diagnostic and therapeutic delay in LOPD patients. A diagnostic algorithm is proposed to facilitate the diagnosis of LOPD in patients presenting with unexplained proximal/axial weakness (with or without respiratory symptoms) or restrictive respiratory insufficiency with hyperCKemia and/or exercise intolerance as the red flag symptoms/signs that raise the index of suspicion for LOPD diagnosis. The diagnosis is based on the subsequent use of dried blood spot (DBS) assay, and the DBS assay can be confirmed by acid alpha-glucosidase (GAA) tissue analysis in leukocytes, fibroblasts, or muscle fibers and/or genetic mutation analysis. Accordingly, experts consider increased awareness among physicians about potential presenting characteristics with a high index of suspicion for LOPD to be crucial to suspect and consider LOPD in the differential diagnosis, while strongly suggesting the use of a diagnostic algorithm combined with DBS assay and confirmatory tests in the timely diagnosis of LOPD and implementation of best practice patterns.

## Introduction

Pompe disease (PD), also known as glycogen storage disease type II (GSDII) or acid maltase deficiency (AMD), is a rare lysosomal storage disorder caused by a genetic deficiency of acid α-glucosidase (GAA) enzyme which results in the accumulation of lysosomal and non-lysosomal glycogen and the alteration of autophagy and cell signaling in multiple tissues, primarily in the muscle tissue (1–3).

PD has a broad spectrum of clinical manifestations depending on the age of onset, progression rate, and genetic mutations (2, 4). Accordingly, PD is classified as infantile-onset PD (IOPD; no residual GAA activity) associated with generalized hypotonia, cardio-respiratory failure, and mortality in the 1st year of life and late-onset PD (LOPD; residual levels of GAA activity), which is further classified into juvenile-onset PD (JOPD) and adult-onset PD (AOPD) that presents at any time after the 2nd year of life and is associated with a less severe phenotype involving progressive limb muscle weakness often mimicking limb-girdle muscular dystrophies (LGMD) or inflammatory myopathies, respiratory insufficiency, and only rarely with cardiac involvement (2, 4–8).

Progressive proximal and axial muscle weakness leads to problems with activities of daily living (ADL), significant motor disability, reduced mobility, and eventual wheelchair use, while respiratory muscle involvement leads to respiratory distress mainly due to diaphragmatic weakness and respiratory insufficiency that is the primary cause of morbidity and mortality in LOPD patients (2, 9–14).

Given the rarity and wide clinical spectrum of the disease which manifests with initially non-specific symptoms and a highly variable course, a high index of suspicion is needed to recognize LOPD in clinical practice (15, 16). Accordingly, along with the lack of awareness and recognition of the disease among physicians, LOPD diagnosis remains a challenge with high rates of poor recognition, underdiagnosing, and substantial diagnostic delay (15–18). Hence, the disease may remain undiagnosed for many years despite the likelihood of a simple screening of the disease via enzyme levels in dried blood spots (DBS) in suspected cases, leading to severe complications that are otherwise preventable or reversible by enzyme replacement therapy (ERT) (17, 19–24).

Early referral of patients with unspecific symptoms to expert centers is considered an improved strategy to facilitate early diagnosis of PD (15). There are a limited number of neuromuscular expert centers in Turkey, necessitating the increased awareness of LOPD among general neurologists, as well as in other most consulted physicians (physical therapists, general practitioners, and orthopedists) to prevent missed cases, enable timely diagnosis, and reduce the risk of lack of access to timely and appropriate medical care in this patient population (15, 17, 25). Hence, given the availability of enzymatic and/or blood-based genetic tests in case of clinical suspicion, the development of algorithms to promote timely diagnosis of LOPD that can guide clinicians is considered to be of critical importance (13, 17, 22–24).

The proposed expert opinion was therefore prepared by a panel of neurology specialists experienced in neuromuscular diseases from Turkey to review the current knowledge on LOPD and to provide a practical and implementable guidance document to assist clinicians with best clinical practice in terms of diagnosis, treatment, and monitoring of patients with LOPD.

## Methods

The present expert panel of neurology specialists with long-term experience in LOPD management met to develop an expert opinion on the diagnosis and management of LOPD from neurologists' perspective to facilitate the diagnosis of LOPD for the neurologist. All experts were informed about the study via e-mail by the sponsor (Sanofi Turkey) and then asked to participate in two consecutive board meetings supported by the sponsor to achieve the proposed opinion. A literature search was performed via PubMed (January 2004–April 2022 inclusive) using the keywords “LOPD, Pompe disease, diagnosis, clinical presentations, algorithm, treatment, and enzyme replacement therapy” aligned with the strategy, while additional publications were also added through citation tracking. The panel critically analyzed recommendations from international guidelines and consensus statements, systematic reviews, results of randomized control trials, population-based studies, prospective longitudinal cohort studies, multicenter cross-sectional studies, and case reports focusing on LOPD and agreed on a series of statements supported by scientific evidence and expert clinical opinion to assist clinicians in real-life practice. The proposed expert opinion planned to provide a practical and implementable guidance document addressing the approach to LOPD in terms of (a) clinical manifestations, (b) diagnostic odyssey (diagnostic delay and proposed diagnostic algorithm), and (c) treatment (ERT, treatment response, endpoints, limitations of ERT, and future therapies) of the disease.

## Clinical manifestations of LOPD

LOPD is a multisystem disorder with variable severity, manifesting initially with asymptomatic hyperCKemia, exercise intolerance, fatigue, or myalgia, and progressing to a symptomatic limb-girdle and axial weakness and respiratory insufficiency due to diaphragmatic and intercostal muscle weakness (13, 24, 26). A summary of LOPD clinical multisystem involvement and related differential diagnosis is provided in Table 1.

**Table 1:** LOPD clinical multisystem involvement and related differential diagnosis (13, 24).

**Organ involvement**	**Clinical manifestations**	**Differential diagnosis**	**Shared signs/ symptoms**
Skeletal muscle	Exercise intolerance/fatigue Myalgia/hyperCKemia Axial and proximal muscles weakness Scapular winging	Limb–girdle muscular dystrophy (LGMD)	Progressive muscle weakness in the pelvis, legs, and shoulders; elevated creatinine kinase (CK)
		Becker muscular dystrophy (BMD)	Progressive proximal muscle weakness, prominent quadriceps weakness, calf hypertrophy, elevated CK
		Myasthenia gravis	Proximal muscle weakness
		Spinal muscular atrophy	Progressive proximal muscle weakness and atrophy, mild elevated CK
		Polymyositis	Unexplained muscle weakness with elevated CK
		Glycogen storage diseases: IIIa (Debrancher deficiency/Cori), IV (branching enzyme deficiency/Anderson disease), V	Hypotonia, muscle weakness with distal involvement, elevated CK
		Danon disease	Skeletal muscle myopathy
		Mitochondrial myopathies	Hypotonia, muscle weakness, elevated CK
		Lipid storage myopathies	Fluctuating muscle weakness, elevated CK
		Selenoprotein N1-related myopathy	Muscle hypotrophy
Respiratory	Morning headache and Sleepiness Sleep apnea Shortness of breath Impaired cough Dyspnea (more at supine position)	Selenoprotein N1-related myopathy	Respiratory failure
		Spinal muscular atrophy	Respiratory failure
		Lipid storage myopathies	Respiratory involvement
Musculoskeletal-bone	Osteopenia/osteoporosis Vertebral fractures Rigid/bent spine syndromes Scoliosis/kyphosis/hyperlordosis	Selenoprotein N1-related myopathy	Spinal rigidity
Central nervous system/cerebrovascular system	Vertebrobasilar dolichoectasia Intracranial aneurysms Stroke Cerebral hemorrhages Lacunar encephalopathy Sensorineural deafness	Myasthenia gravis	Ptosis, ophthalmoplegia, bulbar dysfunction,
		Mitochondrial myopathies	External ophthalmoplegia
Vascular system	Dilated arteriopathy Aortic stiffness Thoracic and basilar aortic aneurysms		
Cardiac	Rhythm disturbances Cardiac hypertrophy	Becker muscular dystrophy (BMD)	Cardiomyopathy
		Danon disease	Hypertrophic cardiomyopathy
		Mitochondrial myopathies	Hypertrophic cardiomyopathy
Gastrointestinal	Macroglossia Dysphagia Early satiety Chronic diarrhea	Glycogen storage diseases: IIIa (Debrancher deficiency/Cori), IV (branching enzyme deficiency/Anderson disease), V	Hepatomegaly and hepatic failure

Musculoskeletal involvement in LOPD is dominated by progressive muscle weakness affecting the proximal more than distal muscles (20, 27, 28). Proximal lower limb and paraspinal trunk muscles (difficulties in walking, running, performing sports, climbing stairs, or standing up from the floor, bed, or chair) usually are affected first, followed by further involvement of skeletal muscles and respiratory muscles (dyspnea, obstructive sleep apnea, recurrent pneumonia, morning headache, and excessive daytime sleepiness), particularly the diaphragm and the intercostal and accessory muscles (13, 26, 29–33). Subsequent secondary musculoskeletal complications include contractures, limb and spinal deformities (winging scapula, scoliosis, lumbar hyperlordosis, and rigid spine syndrome), and osteopenia/osteoporosis (20, 23, 27).

The cardiac involvement, often present in IOPD, is usually absent or very mild in LOPD being characterized by cardiac arrhythmias, ventricular hypertrophy, and Wolf-Parkinson-White syndrome (7, 31). Recent studies have also reported several clinical manifestations related to the involvement of different organs in LOPD such as facial and bulbar weakness (tongue weakness, swallowing disturbances, dysphagia, and dysarthria), ophthalmologic abnormalities (eyelid ptosis, strabismus, and less frequently ophthalmoplegia), sensorineural hearing impairment, vascular abnormalities with cerebral aneurysms, gastrointestinal involvement with macroglossia, hepatomegaly, diarrhea and low body mass index (13, 31, 34–36).

A restrictive respiratory insufficiency, mainly due to diaphragmatic weakness, may be the first presentation of LOPD as evident before any other significant weakness so patients may have respiratory disorders despite retaining ambulation (29, 33, 37, 38).

## Diagnostic odyssey of LOPD

### Diagnostic delay

Due to non-specific symptoms that overlap with many other neuromuscular disorders, the rarity and wide clinical spectrum of the disease as well as the insufficient awareness among physicians remain a challenge for detecting patients with LOPD in clinical practice, therefore misdiagnosis or delayed diagnosis of LOPD is frequent (16, 31). The early diagnosis is relevant due to the likelihood of improving or at least stabilizing the course of the disease through ERT (19, 39).

The diagnostic delay due to the heterogeneous presentation has been reported to range from 5 to 30 years from the onset of symptoms (10, 18, 40), while almost one-third of patients are considered to receive incorrect diagnoses before seeing a metabolic or neuromuscular expert, including “unclear muscle dystrophy/ hypotonia/weakness” and “ankylosing spondylitis/degenerative back disease” in most cases (15).

Although the combination of limb-girdle muscle weakness with respiratory distress is a red flag for LOPD, the time lapse between the onset of symptoms and establishment of diagnosis in LOPD is quite delayed probably due to the insidious onset of an ordinary limb-girdle weakness with unexceptional features and the insidious onset of respiratory insufficiency, which might be tolerated by the patient for many years (17, 41, 42). Indeed, the so-called “respiratory phenotype” is considered a likely confounder in the delayed diagnosis of LOPD diagnosis (23, 43).

### Raising the index of suspicion: a proposed diagnostic algorithm

Due to wide variation in age of onset and non-specific symptoms that can clinically resemble a myriad of other neuromuscular disorders, the diagnosis of LOPD is often challenging, necessitating a high level of clinical suspicion for a timely and accurate diagnosis (24, 27).

In LOPD, the first clinical manifestation can be either proximal muscle weakness or other complaints such as exercise intolerance, muscle pain, or even isolated hyperCKemia, which are similar to those in other hereditary or acquired muscle disorders (i.e., LGMD, other muscle glycogenosis, and inflammatory myopathies) (44, 45).

Accordingly, the suspicion of the disease by clinicians is the key factor in establishing the diagnosis of rare diseases, and is particularly important for LOPD, given the availability of convenient blood-based enzymatic diagnostic testing and genetic sequencing (17, 19, 39, 46).

**Figure 1:**
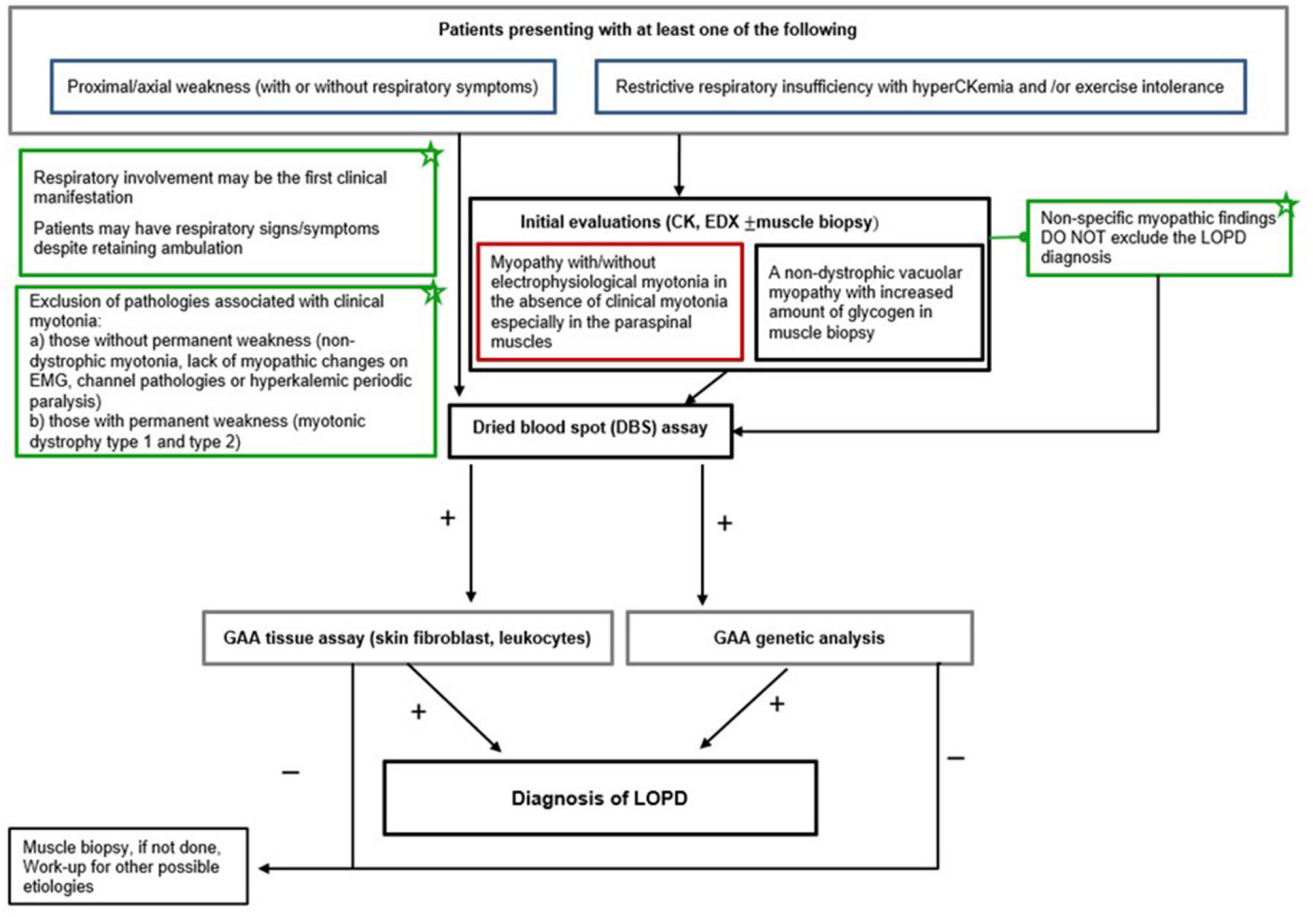
LOPD diagnostic algorithm. EDX, electrodiagnosis; DBS, dried blood spot; LOPD, late-onset Pompe disease; EMG, electromyography; GAA, acid alpha-glucosidase; CK, creatine kinase.

Figure 1 displays the proposed diagnostic algorithm as per the experts' recommendations to facilitate the diagnosis of LOPD presenting with the red flag symptoms/signs. Accordingly, the experts recommend that after clinical history, neurological examination, and routine laboratory tests, the presence of unexplained proximal/axial weakness (with or without respiratory symptoms) or restrictive respiratory insufficiency with hyperCKemia (up to 15-fold) and/or exercise intolerance should be considered as the red flag symptoms/signs that raise suspicion for LOPD diagnosis (Figure 1). The next step should be electrophysiological studies (comprehensive needle electromyography, motor nerve conduction velocities) to address different neuromuscular disorders (31). The presence of electrophysiological myotonia in the absence of clinical myotonia and permanent weakness, particularly in paraspinal muscles, are highly suggestive of LOPD diagnosis (20, 47). Nonetheless, it should be noted that normal CK values or the normal findings on EMG or muscle biopsy do not exclude the LOPD diagnosis (13, 18, 20, 31, 47) (Figure 1).

In patients with a suspected diagnosis of LOPD, subsequent use of DBS to test the GAA enzyme activity reveals the diagnosis, while the DBS assay can be confirmed by acid alpha-glucosidase (GAA) tissue analysis in leukocytes, fibroblasts, or muscle fibers and/or genetic mutation analysis (24) (Figure 1).

## Treatment of LOPD

Late-onset Pompe disease is a multisystem disorder that requires the involvement of a multidisciplinary team to properly treat the pulmonary, neuromuscular, orthopedic, and gastrointestinal elements of the disease (20, 27).

### Enzyme replacement therapy

The development of the ERT considerably changed the prognosis of PD, and this treatment modality represents the current standard of care for the disease. The use of ERT in LOPD has been approved in Europe since 2006 and in the USA since 2010 (alglucosidase alfa, Lumizyme^®^ within the USA, Myozyme^®^ outside of the USA, Nexviazyme^®^ [by FDA in 2021], and Nexviadyme^®^ [by EMA in 2022], Sanofi) and based on 20 mg/kg body weight dosage every 2 weeks as an intravenous infusion (48, 49).

After a diagnosis of LOPD by DBS assay and a confirmatory secondary test such as enzyme testing or genetic studies, the decision to start treatment with alglucosidase alfa should be made depending on the status of the patient (20, 24).

- Patients symptomatic at diagnosis with demonstrable muscle weakness on physical examination or reduction in pulmonary parameters on pulmonary function testing should begin treatment with ERT immediately, and treatment is recommended regardless of the use of non-invasive ventilation (20).
- Presymptomatic patients with objective signs of LOPD including proximal muscle weakness detectable on manual muscle testing or reduction in respiratory parameters, as evidenced by reduced forced vital capacity (FVC < 80%) should be treated with ERT (20, 49).
- Presymptomatic patients without symptoms or signs should be observed without the use of ERT and monitored every 6 months in terms of evidence of clinical deterioration in muscle strength or pulmonary functions (assessed by muscle strength tests or daily living activity evaluations) to initiate ERT (20, 24). This group also includes patients with fatigue or myalgia, elevated CK levels, and minimal pathological findings in muscle imaging or biopsy without muscle weakness or respiratory involvement. ERT is recommended at the earliest onset of objective signs of PD (20, 49).
- In patients with markedly advanced disease who have lost ambulation and are ventilation-dependent, ERT should be administered for 1 year with the evaluation of effectiveness, and after 1 year, the decisions regarding the continuation of ERT should be made on a case-by-case basis with the continuation of ERT in patients who display a stabilization or improvement in symptoms (20, 24).

European consensus guidelines on the use of ERT in LOPD recommend reconsidering whether ERT should be continued if skeletal muscle function or respiratory function has not stabilized or improved in the first 2 years after the start of treatment (46). However, there are still unresolved problems to monitor treatment response and disease progression in Pompe disease. Patients should be monitored every 6 months. The minimal clinical assessment set should include manual muscle testing according to the Medical Research Council grading scale, a 6-min walk test, and timed tests (10 m walk test, time to climb four stairs, and stand up from spine position and from the chair), and it is even better to do the quick motor function test. Pulmonary functions should be assessed with forced vital capacity measurement preferably in supine and sitting positions.

Although there has been considerable work done to validate muscle MRI as an outcome measure, the number of available examinations is limited. In non-responders, treatment should be discontinued and monitoring should be continued with restarting ERT if the rate of deterioration increases after discontinuation of ERT (24).

### Treatment response—Endpoints

In untreated LOPD patients, minimal clinically important differences for deterioration in FVC would occur in approximately 2 years and deterioration in FVC would occur in the 6-min walk test (6MWT) within 9 years (22). In treated LOPD patients, the initial positive response to ERT is mostly followed by a slow and seemingly linear decline along with considerable variability among patients in treatment response (good response lasted up to 7 to 8 years in some patients while a secondary decline was observed after 1 to 2 years in others) and in certain outcomes in the same patient (clear improvement in walking ability while deteriorating in pulmonary function or vice versa), challenging the prediction of the timing in the change in responsiveness (50).

The alglucosidase alfa treatment has consistently been reported to be associated with improved walking distance and ambulation maintained over time, prevention of deterioration in respiratory function, and stabilized or improved CK levels and muscular and/or respiratory function along with an increased life expectancy and survival (6, 19, 21, 24, 51).

Although significant clinical benefits have been attained with the standard of care ERT alglucosidase alfa, there typically is a clinical plateau or a decline over time and most patients with LOPD eventually progress to physical debilitation requiring the use of a wheelchair and assisted ventilation, with premature death often occurring due to respiratory failure (29, 30).

Overall, the factors (i.e., type of the disease causing the mutation, the baseline status of the disease, the lifestyle, and the diet of the patient) underlying the great variability in treatment response, which is only partially associated with the antibody titer against the therapeutic protein, have not yet been clarified and predicting the responders and non-responders before treatment initiation is therefore not possible (50, 52–55).

Hence, the adjunctive use of endpoints based on patient-reported outcome measures, in conjunction with quantitative clinical assessments, may provide a substantial body of evidence to support the conclusion that a treatment or a drug is providing clinical benefits (30).

### Limitations of ERT-future therapies

Although >90% of patients benefit from ERT for the first 3 to 5 years, the observed secondary decline, suggesting diminished therapeutic efficacy over time, raises concerns and stresses the need for next-generation therapies (50).

Indeed, the ERT doses used in LOPD are markedly higher than those required in other lysosomal storage disorders, possibly reflecting the higher threshold for the correction of GAA deficiency in the skeletal muscle of Pompe patients (48). In addition, the liver takes up most of the recombinant human GAA (rhGAA) (up to 85%) and considerably limits muscle targeting, while the inability of the recombinant enzyme to cross BBB limits nervous system efficacy (48).

Another important shortcoming of ERT is related to its limited efficacy in terms of respiratory function (approximately 30% of treated patients end up requiring assisted ventilation, either invasive or not, over the course of their life) and quite a variable improvement in skeletal muscle function (from maintenance of independent ambulation to minor improvements with the eventual wheelchair-bound state) (56–59).

Nonetheless, efforts were dedicated to overcoming some of the limitations of the treatment including those aimed at the enhancement of the enzyme bioavailability in tissues (modification of the recombinant enzyme to increase the mannose-6-phosphate [M6P] residue content and use of pharmacological adjuvants to enhance ERT efficacy, use of chimeric GAA proteins carrying uptake domains to enhance clearance of glycogen, and use of chaperones to enhance enzyme stability in the blood) (48).

Avalglucosidase alfa (Nexviazyme, Sanofi Genzyme, Cambridge, MA, USA) is a rhGAA ERT specifically designed for enhanced M6P receptor (M6PR) targeting and enzyme uptake aimed at increased glycogen clearance and received FDA approval for the treatment of LOPD in August 2021 (60). Recently, the phase 3 COMET trial in 100 treatment-naïve patients with enzymatically confirmed LOPD who were randomly allocated avalglucosidase alfa (*n* = 51) or alglucosidase alfa (*n* = 49) revealed that avalglucosidase alfa improved the upright FVC% predicted by 2.89% (SE 0.88) compared to 0.46% (0.93) with alglucosidase alfa at week 49, showing the non-inferiority of avalglucosidase alfa (difference 2.43% [95% CI 0.13 to 4.99]) (60). The authors also noted improved 6 MWT with avalglucosidase alfa compared with alglucosidase alfa, with a greater increase in distance covered (difference 30.01 m [95% CI 1.33 to 58.69]) and percent predicted (4.71% [0.25 to 9.17]) as well as a more favorable safety profile (serious treatment-emergent adverse events in 16% vs. 25% of patients and infusion-associated reactions in 26% vs. 33% of patients, respectively) (60). Hence, avalglucosidase alfa therapy is considered to provide better outcomes over alglucosidase alfa even though testing for superiority was borderline significant (*p* = 0.0626) (60).

Another novel agent cipaglucosidase alfa in combination with an enzyme stabilizer miglustat was tested in a double-blind phase 3 trial and compared with alglucosidase alfa in 117 LOPD patients who were randomly assigned to intravenous cipaglucosidase alfa (20 mg/kg) plus oral miglustat or intravenous alglucosidase alfa (20 mg/kg) plus oral placebo once every 2 weeks for 52 weeks (61). The authors reported that cipaglucosidase alfa plus miglustat did not achieve statistical superiority to alglucosidase alfa plus placebo for improving 6-min walk distance [mean (SE) change from the baseline in 6-min walk distance at week 52 was 20.8 (4.6) m and 6.2 (6.6) m, respectively] (61).

Although the superiority of next-generation enzyme treatments over alglucosidase alfa could not be demonstrated, the fact that better results have been obtained with these new agents, while statistically not significant, still makes them reasonable as initial treatments in newly diagnosed patients until we see the long-term results. Moreover, in patients who showed a secondary decline with alglucosidase alfa, or in patients with side effects or allergic reactions to alglucosidase that limit treatment, change to next-generation therapies should be considered (60, 61).

Furthermore, given its monogenic origin, PD represents an ideal target for the development of gene replacement strategies and gene therapy, therefore, holds the potential to revolutionize the way we treat PD, virtually providing a steady state supply of GAA enzyme to the entire body following a single medical intervention (48, 62).

Notably, concomitant follow-up for regular exercise and nutrition status is also suggested in LOPD patients, while prescription of a dietary (low carbohydrate–high protein diet) and aerobic exercise protocol concomitant to ERT is considered a beneficial supportive complementary strategy likely to improve ERT response rates (63).

## Conclusion

Owing to non-specific symptoms that overlap with many other neuromuscular disorders, the rarity and wide clinical spectrum of the disease as well as the insufficient awareness among physicians, identifying patients for LOPD diagnostic testing remains a challenge in clinical practice, and misdiagnosis or delayed diagnosis of LOPD is frequent despite the chance of improving or at least stabilizing the course of disease through ERT. The participating neurology experts consider the clinical suspicion of LOPD by the physician to be of utmost importance in the prevention of diagnostic and therapeutic delay in LOPD patients and the importance of addressing the potential presenting characteristics with a high index of suspicion in terms of implementation of best practice patterns. The experts strongly suggest the use of a diagnostic algorithm combined with DBS assay, GAA tissue analysis in leukocytes, fibroblasts, or muscle fibers, and/or genetic mutation analysis in the timely diagnosis of LOPD in patients presenting with unexplained proximal/axial weakness (with or without respiratory symptoms) or restrictive respiratory insufficiency with hyperCKemia who had electrophysiological myotonia in the absence of clinical myotonia, particularly in paraspinal muscles on electrophysiological assessments. The experts also consider the likelihood of an improved clinical response to treatment with early initiation of ERT as well as the clinically meaningful improvement in respiratory function, ambulation, and functional endurance and, thus, a longer stabilization period with the use of novel rhGAA ERT (avalglucosidase alfa) specifically designed for enhanced M6PR targeting and enzyme uptake in patients with LOPD. In conclusion, this consensus statement by a panel of neurology experts provides a practical and implementable guidance document to assist clinicians in best clinical practice in terms of diagnosis, treatment, and monitoring of LOPD.

## Data availability statement

The original contributions presented in the study are included in the article/supplementary material, further inquiries can be directed to the corresponding author.

## Author contributions

All authors listed have made a substantial, direct, and intellectual contribution to the work and approved it for publication.
